# Airway Epithelium Interactions with Aeroallergens: Role of Secreted Cytokines and Chemokines in Innate Immunity

**DOI:** 10.3389/fimmu.2015.00147

**Published:** 2015-04-02

**Authors:** Vivek D. Gandhi, Harissios Vliagoftis

**Affiliations:** ^1^Pulmonary Research Group, Department of Medicine, University of Alberta, Edmonton, AB, Canada

**Keywords:** airway epithelium, proteinases, pollutants, airway inflammation, HDM allergens, cockroach allergens, fungal allergens

## Abstract

Airway epithelial cells are the first line of defense against the constituents of the inhaled air, which include allergens, pathogens, pollutants, and toxic compounds. The epithelium not only prevents the penetration of these foreign substances into the interstitium, but also senses their presence and informs the organism’s immune system of the impending assault. The epithelium accomplishes the latter through the release of inflammatory cytokines and chemokines that recruit and activate innate immune cells at the site of assault. These epithelial responses aim to eliminate the inhaled foreign substances and minimize their detrimental effects to the organism. Quite frequently, however, the innate immune responses of the epithelium to inhaled substances lead to chronic and high level release of pro-inflammatory mediators that may mediate the lung pathology seen in asthma. The interactions of airway epithelial cells with allergens will be discussed with particular focus on interactions-mediated epithelial release of cytokines and chemokines and their role in the immune response. As pollutants are other major constituents of inhaled air, we will also discuss how pollutants may alter the responses of airway epithelial cells to allergens.

## Introduction

Allergic asthma is a complex disease that involves interactions of genetic and environmental factors (1). Genetic factors predispose to atopy, but the development of allergic sensitization is also dependent on environmental factors with exposure to a particular allergen being one of the most important. When sensitized individuals are exposed to the same allergen, they develop allergy symptoms from target organs; these symptoms can be organ specific in conditions like allergic rhinitis and asthma, or generalized, as in the case of anaphylaxis.

Aeroallergens are the major triggers for respiratory allergy (2), although foods, drugs, and other allergens can also trigger disease. The major mode of entry of aeroallergens in the body is through inhalation, although these allergens can also affect the immune system following skin exposure. The airway epithelium is the first line of defense against inhaled aeroallergens (3). The epithelium acts as a structural barrier to prevent invasion of inhaled particles carrying aeroallergens, but it is also the first innate immune cell type that interacts with antigens and other components of the inhaled particles. Studies on these interactions support the idea that epithelial innate immune functions may be instrumental for the development of an immune response through pro-inflammatory mucosal responses, following interactions with inhaled antigens and chemicals found in pathogens, allergens, and pollutants.

The theme of the review will be the role of epithelium as innate immune cells in allergic airway inflammation. Our discussion will focus primarily on cytokines and chemokines released upon interaction of the airway epithelium with major aeroallergens and how these mediators shape the immune response toward the inhaled allergen. We will also discuss the role of air pollutants in modifying the results of these interactions.

## Airway Epithelium: An Immunologically Active Barrier

Airway epithelial cells express tight junction (TJ) proteins such as occludin, claudin, and zonula occludens, which give epithelial cell monolayers their barrier property. Apart from being a barrier, the airway epithelium plays multiple roles, such as maintaining airway surface liquid (ASL) levels, the mucociliary escalator, and also epithelium restitution upon injury (4, 5). To fulfill these roles, the airway epithelium is comprised of a number of specialized cell types that work in harmony to maintain homeostasis in the airways. The characteristics of the various epithelial cell types found in the lungs and their specific roles are summarized in Table 1.

**Table 1:** **Different airway epithelial cell types and their characteristics**.

	Cell type	Characteristics/functions
**Bronchial Epithelium**	Basal cells	• Only cells that express hemidesmosomes, thus firmly attached to the basal membrane via integrins
		• Self-renewal capacity
		• Act as progenitor for goblet and ciliated cells
		• Produce variety of bioactive molecules including cytokines
	Columnar ciliated cells	• Terminally differentiated cells that arise from either basal or goblet cells
		• Possess cilia that clear mucus from the airways
	Goblet cells	• Secrete mucus into the airways to trap foreign particles
		• Self-renewal capacity
		• Transdifferentiate into ciliated cells
	Club cells	• Produce bronchiolar surfactant and specific antiproteinases, such as secretory leukocyte proteinase inhibitor and other enzymes
		• Progenitor for goblet and ciliated cells

**Alveolar Epithelium**	Type I cells	• Very thin cells that cover 97% of alveolar place
		• This thin structure is important as it allows easy gas exchange between alveoli and blood
	Type II cells	• Produce pulmonary surfactants that are important for keeping alveolar space open and thus allow gas exchange
		• Progenitor cells for alveolar epithelium

Identification of pattern recognition receptors (PRRs) (6) and proteinase-activated receptors (PARs) (7) highlighted the potential of airway epithelial cells to sense/interact with allergens. Further secretion of immune mediators through activation of these receptors gave new insight to airway epithelial cells being an immunologically active innate immune cell. Similar to the airway epithelium, epithelium in other organs, such as gut (8, 9) and skin (10, 11), play the same multiple roles in tissue homeostasis and in the development of immune responses to foreign antigens.

Allergen–airway epithelium interactions and their effects on epithelial properties as well as on the immune system will be discussed below.

## Allergen–Airway Epithelium Interactions

The airway epithelium and allergen–epithelium interactions play a pivotal role in airway immune responses. Allergen–airway interactions start with the recognition of an allergen by receptors present on the airway epithelium. These interactions result in partial loss of epithelial integrity and/or release of inflammatory mediators from epithelial cells. Inflammatory mediators can activate the innate immune system at the same time that the allergen can interact and activate dendritic cells (DCs) that are present below the epithelial monolayer. The default response of immune system to most of these interactions is the development of immune tolerance, which means that subsequent interactions of the organisms with the same allergen will not lead to pathology. In certain individuals, however, the same interactions can lead to the development of allergic sensitization. The exact circumstances and factors responsible for the decision between tolerance and allergic sensitization are not well understood. In mouse experimental models, it has been shown that activation of PRRs (12) or PAR-2 (13) can bias the system toward sensitization. It is also known that inflammatory mediators produced by epithelial cells after interactions with allergens may bias the immune response toward allergic sensitization through their effects on allergen-DC-T cell interactions (3). A subsequent exposure of an individual with an allergic sensitization to the same allergen results in IgE-mediated activation of mast cells (14). However, even in sensitized individuals, inflammatory mediators released from epithelial cells following interactions with allergens play an important role in recruiting inflammatory cells and mediating allergic inflammation. Since the aim of the review is to discuss the role of allergen–epithelium interactions and its immune outcomes, we will focus on the epithelial responses that play role in allergic sensitization, the first part of the process described above, and allergic inflammation, the second part of the process.

The airway epithelium–allergen interactions and the functional consequences of these interactions are affected by the structural and functional state of the epithelium at the time of these interactions. For example, decreased TJ protein expression in atopic asthmatics (15) could compromise the epithelial barrier function (16) allowing allergen invasion. Moreover, there is evidence that mucociliary clearance is not efficient in asthmatic airways (17, 18). It is not clear whether this impairment is the result of environmental factors, including the effects of inhaled allergens, or driven by genetics, but ineffective airway clearance may result in prolonged presence of the allergens in the airways allowing them to have protracted effects. In addition allergen can directly alter epithelial properties, as will be discussed below.

*In vitro*, *ex vivo*, and *in vivo* systems have been used to study the outcomes of direct allergen–epithelium interactions. *In vitro* approaches have generated interesting results about epithelial responses to allergens, but the biological relevance of these results is questionable as these interactions happen under artificial conditions and in the absence of other cells/factors, such as other immune cells that may modulate the effects. *Ex vivo* and *in vivo* approaches on the other hand suffer from the inability to determine that the effects seen are mediated by direct epithelium–allergen interactions and are not due to the indirect effects of other immune cells–allergens interactions on the airway epithelium. However, the two approaches give complementary and very important results and we will review them separately below.

*In vitro* research to understand allergen–epithelial interactions has been carried out using both alveolar and bronchial airway epithelial cells. A549 is the most utilized cell line to study interactions with alveolar epithelium while various cell lines, such as BEAS-2B, 16HBE14o-, Calu-3, NCI-H292, and primary airway epithelial cultures, from healthy and asthmatic patients, have been used to study interactions with the bronchial epithelium. In addition, both immersed cultures and air–liquid interface (ALI) cultures have been used in studies performed with bronchial epithelial cells. ALI cultures mimic a physiological airway epithelium and facilitate *in vitro* study of epithelial functions such as barrier property, mucus secretion, and mucociliary escalator, which is not possible with immersed cultures. ALI cultures also provide a unique opportunity to study cellular interactions between epithelial cells and other immune cells by using co-culture systems. However, this variety of experimental settings and cells used has generated often inconsistent and difficult-to-explain results.

**Figure 1:**
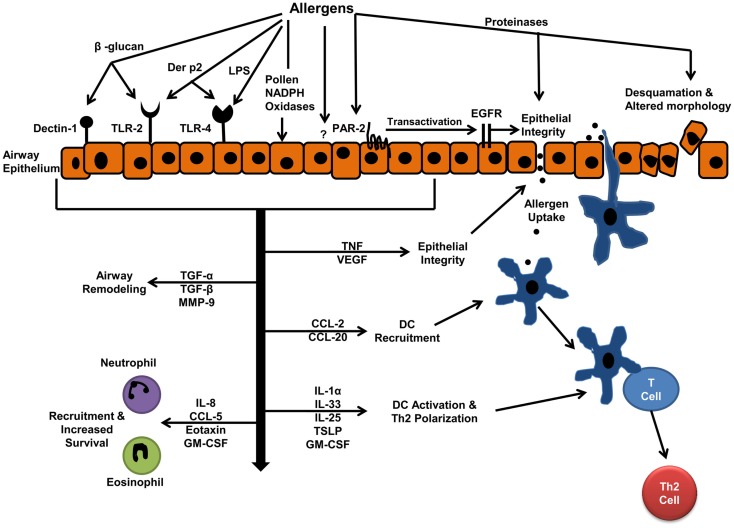
**Allergen–epithelial interactions: effects on allergic sensitization, and allergic airway inflammation**. Aeroallergen interactions with epithelium result in increased epithelial permeability, which facilitates allergen entry and uptake by dendritic cells (DCs). The mediators released by epithelium, upon allergen interactions, drive DC-T cell interactions toward Th2 immune response. These mediators also develop inflammation by recruiting inflammatory cells to the airways.

In the following section, we will review studies focusing on interactions of airway epithelial cells with some of the most common aeroallergens including house dust mite (HDM), cockroach, fungal, and pollen allergens. A schematic of the mechanism of these interactions and the biological effects of interactions are shown in Figure 1. Allergens include proteins with very different structures and activities and can interact with epithelial cells through a variety of mechanisms. *In vitro* studies have shown the proteinase activity of allergens can alter epithelial cell morphology and cause epithelial cell detachment (19, 20). Moreover, proteinase activity of allergens has been implicated in allergic sensitization and allergic inflammation in animal models (21). In this review, we will classify the allergen–epithelial interactions into proteinase-dependent and proteinase-independent interactions.

### Epithelial tight junction disruption

Most of the direct effects of allergens on the immune system require allergens to penetrate through the epithelium into deeper tissues. The main mechanism by which allergens cross the epithelial barrier is through the degradation of epithelial TJs. A HDM allergen with cysteine proteinase activity can degrade the adhesion protein occludin and allow allergens to penetrate through the epithelial monolayer (22). Similarly, cysteine and serine proteinases from pollen grains (23, 24) and fungi (25) can induce TJ degradation.

In addition to direct degradation of TJ, HDM proteinases can also have an indirect effect on TJ integrity. HDM proteinases activate PAR-2 (26), a pro-inflammatory receptor on epithelium, which in turn transactivates epidermal growth factor receptor leading to E-cadherin destabilization and loss of epithelial barrier integrity (27). Thus, although proteinases associated with allergens or allergen particles are prime candidates to mediate TJ degradation, this may not always be the case. Another study comparing various HDM extracts from different manufactures showed that the extract with the lowest proteinase activity caused maximum barrier dysfunction (28). The study also showed that this extract was the most potent inducer of inflammatory mediator release indicating that these mediators could contribute to epithelial barrier dysfunction. The idea was supported by the observation that cockroach allergens affect epithelial permeability indirectly through the release of vascular endothelial growth factor (VEGF) from epithelial cells (29). Chronic exposure to tumor necrosis factor-α (TNF) also causes barrier dysfunction by disrupting TJs (30). The observation that fungal allergens can induce TNF release from airway epithelial cells (31) indicates that this may be another indirect method utilized by allergens to increase airway epithelial permeability.

Finally, TJ degradation by allergens may also be specific to epithelium from asthmatics. For example, a study has shown that epithelium from only asthmatic individuals and not from healthy individuals was sensitive to fungal allergens-mediated increased epithelial permeability (31). However, it is not clear whether asthma-induced epigenetic changes make the airways of asthmatic individuals sensitive to allergens or it is genetic defects in the TJ proteins that make an individual more sensitive to allergen penetration, sensitization, and asthma development.

### Epithelial release of inflammatory mediators: Cytokines and chemokines

Allergens interact directly with airway epithelial cells and induce the release of inflammatory mediators, including cytokines and chemokines. In most cases, however, the exact type of epithelial cell responsible for the release of mediators is not described. Many of the studies discussed below utilize immersed cultures of airway epithelial cells, which are comprised primarily of basal cells (32); basal cells have been shown to express receptors that can be activated by allergens (6, 33). Thus, we speculate that basal cells may be the main cell type releasing cytokines and chemokines in response to aeroallergen stimulations, although other epithelial cell types may also be involved. There is more information on the responses of epithelial cells, from different anatomical locations, to interactions with allergens and these will also be discussed below. We also discuss interactions of allergens with alveolar epithelial cells. Although allergen particles may not be able to reach the alveolar space, individual allergenic proteins may bind to other smaller particles [i.e., diesel exhaust particles (DEPs)] and reach this area.

#### House dust mite

House dust mite allergens induced the release of CCL20 from human airway epithelial cells, while cockroach allergens do not have the same effect (34); CCL20 release in this study was dependent on the interaction of β-glucan from HDM with dectin-1 on epithelial cells and independent of proteinase activity. β-glucan from HDM also induced CCL20 release from human nasal epithelial cells, but in this case through interactions with TLR-2 (35). Even though HDM-induced CCL20 release from human airway epithelial cells was proteinase-independent, in mouse models of allergic airway inflammation CCL20 release from airway epithelial cells was shown to be proteinase and PAR-2-dependent (36).

Der p2, an HDM allergen without proteinase activity, induced cytokine release from the airway epithelium through TLR-2 (37) and TLR-4 (38) activation. Der p2 is shown to induce reactive oxygen species (ROS) and ROS-mediated nerve growth factor (NGF) release from airway epithelium (39), and NGF was found to be important for the development of asthma features (40). An *in vivo* study, using a model of tissue specific TLR-4 knockout, showed that HDM-mediated allergic inflammation was dependent on the interaction between HDM and TLR-4 on airway structural cells (41). However, in this model, HDM-induced IL-25 and thymic stromal lymphopoietin (TSLP) were not TLR-4 dependent. IL-25 and TSLP are vital mediators for the development of allergic sensitization and are released primarily by epithelial cells. Thus the presence of these mediators, in the absence of epithelial TLR-4, shows a requirement of other receptors on airway epithelial cells for the development of inflammation.

In line with this argument, HDM has shown proteinase-dependent release of IL-25 from bronchial epithelial cells (42). Moreover, a study also demonstrated that the inflammatory property of HDM was mainly dependent on the proteinase activity of HDM extract (19). Der p3 and Der p9, HDM allergens with serine proteinase activity, induced chemotactic mediators CCL11 and granulocyte macrophage colony-stimulating factor (GM-CSF) release from alveolar cells through PAR-2 (43).

#### Cockroach allergens

Cockroach proteinases can also activate PAR-2 (44) and PAR-2-mediated release of CXCL8 from human alveolar (45) and bronchial epithelial cells (46) as well as CCL20 and GM-CSF from mouse tracheal epithelial cells (36). Cockroach allergens can also induce the release of IL-33 (47) and IL-25 (42) from human bronchial epithelial cells, but the role of proteinases in this instance is not clear.

These studies have primarily used HDM and cockroach whole body extracts to study their interactions with epithelial cells. However, humans are exposed to HDM and cockroach frass in their daily lives. It is true that majority of the allergens that are present in frass are also found in whole body extract (48, 49), allowing us to consider results from crude extracts. Antigen characteristics and abundance may also vary to some degree between species (50, 51) and the composition of allergen extracts varies from manufacturer to manufacturer (28, 49). These differences make at times the studies difficult to interpret and impossible to compare.

#### Fungal allergens

*Alternaria alternata* is the best studied fungal species for interactions with epithelial cells. It has been shown to induce proteinase-dependent IL-6 and CXCL8 release from alveolar (20) and bronchial epithelial cells (52); cytokine release from bronchial epithelial cells was shown to be PAR-2-mediated. Apart from these mediators, *A. alternata* has also been shown to induce PAR-2-mediated release of GM-CSF (52) and TSLP (53) from bronchial epithelial cells, as well as release of IL-33 (47) and IL-25 (42). Involvement of proteinases in the release of IL-33 and IL-25 is not clear. *Aspergillus fumigatus* (54) and *Cladosporium herbarum* (20) also induced proteinase-dependent release of IL-6 and CXCL8 from an alveolar cell line. Purified serine proteinase Pen c13 from *Penicillium citrinum* showed to induce CXCL8 in alveolar cell line and primary cells through PAR-1 and PAR-2 activation (55). Similarly, another purified serine proteinase Pen ch13 from *Penicillium chrysogenum* induced CXCL8, prostaglandin E_2_ (PGE_2_), and transforming growth factor-β (TGFβ) from A549 and primary human bronchial epithelial cells (25). Fungal extracts, similarly with HDM and cockroach extracts, possess chitin and it has been shown that chitin can induce CCL2 from a mouse airway epithelial cell line (56) but the effect of chitin on human airway epithelial cells have not been studied.

As discussed, allergens from HDM, cockroaches, and fungi are able to activate PAR-2 receptors, which are expressed throughout the airways. *In vitro* PAR-2 activation has been shown to release mediators from cells of different phenotypes, which are present in the airways, for example, IL-25 (42) and PGE_2_ from bronchial epithelial cells (7), matrix metallopeptidase 9 (MMP-9) from small airway epithelial cells (57), and CCL2 from alveolar epithelial cells (58).

#### Pollens

Pollen grains are complex structures containing proteins with and without enzymatic activity. We saw in the previous section that proteinases released from pollen grains can alter epithelial integrity; however, pollen-mediated release of mediators has been found to be proteinase-independent (19). Pollen extracts induced release of IL-6, CXCL8, GM-CSF (19), and TGF-β (59) from airway epithelial cells was independent of enzyme activity or lipopolysaccharide (LPS). Pollens also induced IL-25 from bronchial epithelial cells (42), but the mechanism of release is not known. Finally, pollen grains contain NADPH oxidases, which increase intracellular ROS in epithelial cells (60), and thus increase oxidative stress.

A number of the studies discussed here have been carried out with purified allergens and others with crude extracts that contain a great number of different allergens. An *in vitro* study compared inflammatory mediators induced by a purified grass pollen allergen Phl p1 (61) and a crude grass pollen extract (GPE) (62) in a human epithelial cell line. Both stimuli induced many common mediators; however, purified Phl p1 induced the release of granulocyte-colony stimulating factor (G-CSF) while GPE induced CCL3, CCL4, and CCL5. In addition to this differential regulation, the most important difference was that the purified Phl p1 upregulated CCL28 and downregulated TSLP, but the opposite regulation was evident for GPE (63). The authors suggested that the whole extract can have other inhibitory or stimulatory components which could be crucial to overcome/maintain the disease phenotype. This observation was supported by an *in vivo* study, which showed that a purified HDM allergen induced mild late asthmatic responses compared to complete allergen extracts that induced severe responses (64). Thus, it is advisable to use whole allergen extract for studies, which will mimic real life conditions.

Furthermore, the concentrations of allergens used for *in vitro* and/or *in vivo* studies may not be close to physiological concentrations encountered by human airway epithelial cells *in vivo*. Since biological responses are often dependent on the concentration of a protein in addition to the affinity of the ligand for its receptors, results seen in studies may not be similar with what would happen *in vivo* in humans.

#### Synergy with other inflammatory mediators

An important question is why aeroallergens do not induce inflammation in non-asthmatic individuals if they can directly activate epithelial cells. One possibility is that the tissue microenvironment where allergen–epithelial cell interactions take place influences the final outcome of these interactions. Asthmatic airways, in contrast to healthy airways, are characterized by the presence of chronic allergic inflammation. The levels of Th2 inflammatory cytokines such as IL-4 (65), IL-13 (66, 67), and fibrogenic mediators, such as TGF-β (68), are increased in asthmatic airways. There is evidence of synergy for HDM allergens with IL-4 and TGF-β to induce release of the Th2 chemoattractant CCL17 from airway epithelial cells (69). HDM also showed synergy with IL-4 and IL-13 for induction of CXCL8 and GM-CSF release from airway epithelial cells (70). Interestingly, IL-4 also enhanced TSLP release in response to *A. alternata* (53), while the Th1 cytokine interferon gamma (IFNγ) inhibited TSLP induction. TSLP plays an important role in allergic sensitization and thus this observation reflects the importance of the tissue microenvironment in the development of immune response. Cockroach allergens acted synergistically with TNF to induce PAR-2-mediated CXCL8 (71) and MMP-9 (72) release from airway epithelial cells. Finally, PAR-2 activation has also shown synergy with IL-4 for TSLP release (53) and with LPS for CXCL8 release and PAR-2 mRNA expression (73).

In addition, cytokines released by epithelial cells after direct activation by allergens may subsequently activate epithelial cells to release other mediators. For example, direct interaction of epithelial cells with allergens caused TSLP release (53), which could in turn activate epithelial cells to release IL-13 (74). Further, IL-13 acted on airway epithelium to induce periostin (75) and CCL11 (76) release. It was also shown that HDM allergens induced TLR4-mediated IL-1α release from human airway epithelial cells, which acted in an autocrine manner to induce dendritic cell (DC) chemoattractants IL-33 and GM-CSF from airway epithelial cells (77). Inflammation could therefore be perpetuated by this “loop effect” that allergens and cytokines/chemokines exert on the airway epithelium.

#### Asthmatic vs. non-asthmatic airway epithelial cells

A second important question is whether there is a difference in the allergen-induced response between asthmatic and healthy airway epithelia. Attempts to address this question have used cells isolated from the airways of asthmatic and non-asthmatic individuals; these cells retain their characteristics after culture *in vitro* (78). According to one study, untreated cells from HDM-allergic patients showed higher basal expression for genes regulating cytokines, chemokines, and growth factors compared to cells from non-allergic subjects (79). This upregulated expression in the absence of stimulus could be because of asthma-induced epigenetic changes in the epithelium, which could result in constant increased amounts of mediator release. Other studies have also shown that HDM allergen-mediated activation of airway epithelial cells from asthmatic patients, and not from healthy individuals, release CCL20 (80) and transforming growth factor-α (TGFα) (70), which can cause DC chemotaxis and cell proliferation, respectively. The latter manuscript has also shown that cells isolated from asthmatic individuals demonstrate a tendency for increased release of CXCL8 and GM-CSF upon HDM stimulation. These differences could be responsible for the altered immune response to allergens seen in the airways of asthma patients.

#### Effect of epithelial phenotype

We have already discussed the phenotypic and functional heterogeneity of airway epithelial cells. This heterogeneity may explain different epithelial responses to allergens depending on the part of the airways where the interaction takes place. There is substantial experimental evidence showing that cells from different compartments of the airways may respond differently to allergens. The HDM allergens Der p2 (81, 82) and Der f2 (82), which have no proteinase activity, induced IL-6, CXCL8, G-CSF, GM-CSF, CCL2, and CCL20 from bronchial epithelial cells, but not from alveolar epithelial cells, while HDM allergens with proteinase activity induced IL-6, CXCL8, and CCL2 release from alveolar epithelial cells (19) and CCL2 release from bronchial epithelial cells (80), but not from nasal epithelial cells (19). In addition, *A. fumigatus* (54) as well as timothy grass and birch pollens (19) showed CCL2 release from alveolar epithelial cells, but not from bronchial epithelial cells. This information may indicate that in the case of inhalation of multiple allergens, different allergens act on the upper and lower airway epithelial cells causing release of a variety of inflammatory mediators. Also receptors activated by allergens show heterogeneity in their pro-inflammatory responses. Small airway epithelial cells demonstrated PAR-2-mediated CCL11 release (83), while PAR-2-mediated activation of bronchial epithelial cells did not induce the release of CCL11 (52, 84).

In conclusion, interactions between aeroallergens and airway epithelial cells are complex events that are influenced by a number of factors as discussed above. A plethora of mediators are released from airway epithelium as a result of these interactions. In the subsequent sections, we will discuss the effects of these mediators on different immune cells, and thus on the immune response.

## Role of Released Mediators in Shaping the Immune Response

As we mentioned above, allergen–airway epithelial cell interactions participate in both the development of allergic sensitization, and in the development of allergic airway inflammation in sensitized individuals. Here, we will describe the role of various epithelial-derived mediators in these two processes.

### Role in allergic sensitization

Tight junction disruption induced by allergens, as has been discussed above, may facilitate antigen penetration and uptake by DCs, an important first step toward allergic sensitization. However, there is evidence regarding mechanisms, other than TJ disruption, which aid allergen uptake by DCs (85, 86). Another crucial factor required for the development of allergic sensitization is the presence of a favorable microenvironment for DCs. Allergen–airway epithelium interaction results in release of mediators, which provide a favorable environment that supports DC maturation, activation, and also directs DCs interaction with CD4 T cells toward Th2 phenotype, resulting in allergic sensitization and allergic inflammation (87). Most of these studies were done with HDM and cockroach allergens. However, these mechanisms could also be applied to pollen and fungal allergens as they also activate TLR-2, TLR-4, Dectin-1, and PAR-2 receptors that are important for HDM and cockroach effects. Some of the studies showing this effect will be reviewed here.

Allergen-mediated activation of PAR-2 on airway epithelial cells could induce CCL2 release (58), while dectin-1 (34) and TLR-2 (35) activation resulted in the epithelial release of CCL20; CCL2 (88) and CCL20 (89) are chemoattractants for immature DCs. This will result in the recruitment of the immature DCs to the airways, facilitating the first step toward the allergic sensitization, i.e., allergen–DC interaction and allergen uptake by DCs. CCL2 has been shown to play a role in the generation of Th2 responses in mouse models of allergy (90) and also of airway hyper-reactivity (91), a consequence of allergic sensitization. It is interesting that bronchial lavage from allergic asthmatics showed increased CCL20 (80), which indicates that this mechanism may be also important *in vivo* in humans.

Mediators released by the epithelium upon allergen interactions, such as TSLP (92), IL-25, and IL-33 (93), act on DCs to induce OX40L expression. This expression of OX40L on DCs is vital for the development of T cells into the Th2 phenotype (94). In mouse models, interaction of OX40L expressing DC with OX40 on T cells resulted in increased expression of IL-4 (95), which induces T cell differentiation to Th2 cells. Recent evidence showed the inability of DCs to induce Th2 responses in the absence of GM-CSF, another epithelial-derived mediator (96), suggesting a role of GM-CSF in the development of a DC phenotype that promotes Th2 response. In addition to its role in the development of antigen-specific Th2 cells, IL-33 may also play a more direct role in the development of humoral immune responses to inhaled aeroallergens, although the exact mechanism is not clear (97). Finally, IL-33 may also have other pro-inflammatory effects in allergic airway inflammation by supporting eosinophil survival (98) and IL-4 and IL-13 release from basophils (99).

In conclusion, proteinase activity of allergens may facilitate entry and uptake of allergens by DCs, while components, with and without proteinase activity, induce a cytokine/chemokine milieu that supports DCs maturation and polarizes their interactions with T cells toward development of Th2 responses.

### Role in allergic airway inflammation

Interactions of allergens with the airway epithelium are the first events that take place after allergen inhalation by an allergic individual. The mediators released from these interactions play an important role in the development of airway inflammation by acting as chemotactic or survival factors for inflammatory cells.

As mentioned previously, CCL2 (88, 100) and CCL20 (89) recruit monocytes or immature DCs to the site of inflammation. DCs can also act as inflammatory cells and promote inflammation in sensitized individuals (101). GM-CSF increases eosinophil survival (83), while CCL11 acts as chemoattractant for eosinophils to the airway (102). Airway epithelial cells also release neurotrophins under allergic inflammatory conditions that increase eosinophil survival (103).

Allergens mediate release of TGF-β (59). TGF-β is increased in the asthmatic airways and its release was further increased after allergen challenge (68). TGF-β has been shown to induce more extensive epithelial-mesenchymal transition (EMT) *in vitro* in airway epithelial cells isolated from asthmatic individuals compared to cells from healthy individuals (104). In addition, TGF-β-treated, but not untreated, airway epithelial cells undergo EMT upon HDM exposure *in vitro* (105). These observations raise the possibility of differential effect of allergens on airways of asthmatics vs. non-asthmatics but their significance has to be validated *in vivo*. Moreover, TGF-β has found to be involved in airway remodeling by inducing airway smooth muscle cell proliferation and increased mucus production (106). Other released mediators from the airway epithelium, such as IL-25 (107) and TSLP (108), are also involved in airway remodeling. Finally, a recent human study showed that neutralizing TSLP not only prevents the allergen-induced increase in exhaled nitric oxide and blood and sputum eosinophils, but also decreases their levels in mild allergic asthmatics (109).

Allergen-induced epithelial release of MMP-9 has not been reported; however, we have shown that activation of PAR-2, which is a target of allergen-proteinases, causes MMP-9 release from airway epithelial cells (57). Increased presence of MMP-9 in sputum has been observed in patients with severe asthma (110). The same study showed that after allergen challenge, MMP-9 activity was significantly increased in severe and mild asthmatics. MMP-9 could be responsible for airway remodeling by degrading extra cellular matrix (ECM).

As discussed, the activation of airway epithelial cells by allergens also releases CXCL8 and IL-6 (19, 20, 45, 81). CXCL8 may be a chemoattractant for eosinophils in allergic individuals (111), but it may also contribute to the neutrophilia seen in the airways in cases of acute asthma exacerbations or severe asthma (112). IL-6 has been found to be upregulated in severe asthma (113), but its role in asthma is uncertain as it possesses both pro- and anti-inflammatory properties (114).

The majority of these mediators released by the epithelium are also released by other immune cells in the airways. Thus, it would be an overstatement to conclude that the epithelial cell is the sole cell type responsible for the above mentioned responses. However, when HDM allergen-mediated epithelial NF-κB activation was inhibited *in vivo*, allergen-mediated inflammatory mediator release, inflammation, and remodeling was significantly reduced (115). This observation depicts that the mediators release by airway epithelium upon allergen-airway epithelium interactions contribute significantly in the development of allergic sensitization and allergic inflammation.

## Interactions between Allergens and Pollutants

Pollutants are a major pro-inflammatory component of inhaled air and constitute a major health concern (116, 117). Pollutants are present in indoor and outdoor environments and can be gaseous such as ozone and nitrogen dioxide or particulate matter such as DEPs and cigarette smoke (CS). Direct interactions of these inhaled pollutants with airway epithelial cells have been discussed quite extensively elsewhere (118–121). More interestingly, the simultaneous presence of pollutants and aeroallergens in the air results in complex interactions between the two. Pollutants exert direct effects on aeroallergens but also alter the host responses to inhaled aeroallergens.

### Pollutant effects on aeroallergens

Indoor and outdoor airborne particles carry aeroallergens. HDM and cockroach allergens are found in general on particles with a median diameter of 10–30 μm (122), while cat and dog allergens are found on particles with 5 μm mass median diameters (123, 124). Suspended particulate matter in homes can carry dog, pollen (125), and cat allergens (126). DEPs, a major outdoor particulate pollutant, have also been shown to bind pollen, dog, cat, and HDM allergens (125, 127). Because of their very fine size, DEPs can facilitate penetration of these allergens into the lungs and therefore increased numbers of pollution particles may increase the amount of allergen interacting with the epithelium.

It has also been shown that DEPs can disrupt pollen particles causing release of allergenic sub-pollen particles. Interestingly, detailed analysis of pollen obtained from areas with pollution showed increased presence of allergenic proteins (128, 129), which resulted in a higher allergenic property of the allergen (128). This may be another mechanism by which pollution increase allergen–epithelial interactions.

Finally, the effect of climate change and pollution on the allergenicity of pollen has been studied. Comparing recent and a decade-old pollen extract, the authors showed that the allergenic potency of the recent pollen extract was higher (130). It was further shown that the recent pollen extract harvested from the urban area had a higher allergenic potency than the recent pollen extract from suburb. Recently, the same authors also demonstrated that the recent pollen extract from an urban area, which has faced the climate change and increased pollution, was more effective at inducing transepithelial permeability and ROS production in the cultured airway epithelial cells (131).

### Pollutant effects on aeroallergen-induced responses

The effects of various pollutants on the development of allergic sensitization and allergic airway inflammation have been an area of intense research in both animal and human systems. The first evidence 30 years ago showed that inhalation of ozone (132, 133) and DEPs (134) increase sensitization to inhaled allergens as measured by the presence of antigen-specific IgE (132) and subsequently resulted in increased anaphylactic sensitivity upon the allergen challenge. Inhalation of suspended particulate matter along with an allergen has shown to act as an adjuvant and increase IgE production (135). Similarly, in a human study, DEPs, when inhaled with allergen, promoted Th2 inflammation and allergen specific IgE (136).

The mechanisms of this priming/sensitization effect of pollutants have been studied in detail. Interestingly, mice exposed to the pollutant nitrogen dioxide prior to allergen exposure developed TLR2, MyD88, and NF-kB dependent sensitization to the allergen, resulting in Th2 inflammation and airway hyper-responsiveness (137). Pollutants such as ozone (138) and nitrogen dioxide (139) can induce maturation of CD11c+ myeloid DC and increase antigen uptake and antigen-presenting activity of DCs. DEPs and ambient particulate matter upregulated TSLP in bronchial epithelial cells (140) and DEP-induced TSLP can promote myeloid DCs maturation (141) and increase OX40L expression (142), favoring Th2 inflammation. These, and possibly others, are some of the mechanisms of increased allergic sensitization in the presence of pollutants.

The effects of pollutants on allergen-induced responses in sensitized individuals have also been studied *in vivo*. Motorcycle exhaust particles (143) and DEPs (144, 145) can increase airway hyper-responsiveness to allergen and allergen-mediated early and late inflammatory responses in different animal models of allergy. Similarly human studies have shown that prior exposure to pollutants such as ozone, nitric dioxide alone or in combination with sulfur dioxide increased bronchial responsiveness to pollen (146) and HDM allergens (147–149) and also amplified airway inflammation (150, 151).

The *in vivo* studies discussed above, however, do not allow us to identify the cells involved in pollutant–allergen interactions. The airway epithelium would be expected to play a significant role in these effects, especially since pollutants can directly activate airway epithelial cells. Nitrogen dioxide increased epithelial permeability and induced leukotriene C_4_ synthesis (152). Moreover, nitrogen dioxide (153), DEPs (154), and ozone (155) induced CXCL8, GM-CSF, and TNF from cultured airway epithelial cells. As was shown for allergen–epithelial cell interactions, ozone and nitrogen oxide released more inflammatory mediators from epithelial cells from asthmatics than those from healthy donors (156). This observation indicates that pollutants induce a different pro-inflammatory environment in the airways of asthmatics compared to non-asthmatics.

Limited data exist regarding the effect of pollutants on allergen–airway epithelium interactions. These studies have been performed primarily with alveolar cell line A549; because of the fine size of allergen-carrying pollutant particles, these particles can reach to alveolar space and therefore may affect alveolar epithelial cells. Studies have shown that exposure to submicron particles and allergen individually or in combination induced alterations in cellular morphology (increased microvilli), functions (increased lysozyme and surfactant-producing multilamellar bodies) (157), and metabolic activity (damage to mitochondria, tonofilaments, and rough endoplasmic reticulum) in lung epithelial cells (158). Sodium sulfite and HDM acted synergistically for detachment of the cells (159), which could cause inflammation and decrease epithelial barrier integrity. Further, exposure of these cells to the combination of pollen grain-Pb (160) and pollen allergens-DEPs (161) caused significant increase in IL-5 mRNA and Th2 cytokines release, respectively, from airway epithelial cells.

In summary, apart from having inflammatory effects on their own, pollutants can increase the concentration, exposure, and allergenic property of aeroallergens. Thus, pollutants exert a priming effect on immune system for allergens and also increase inflammatory responses to allergens.

Apart from these pollutants discussed so far, CS is a major factor influencing allergic sensitization and asthma. Smoking is a major source of indoor particles (162). Maternal smoking has been shown to increase the risk of asthma development in children (163). Using animal models, it has been shown that passive smoking acts as an adjuvant to increase allergen-mediated allergic immune responses (164) and airway remodeling (165). Effects of CS on airway epithelial cells are similar to other pollutants, i.e., increased allergen-mediated epithelial permeability and inflammatory properties, which have been reviewed in detail (166, 167).

## Conclusion

Current literature establishes the airway epithelium as an innate immunity organ that senses inhaled allergens through an armory of receptors, and initiates innate and adaptive immunity. This potential has been established clearly through *in vitro* studies, although more detailed *in vivo* studies are still needed to validate these results.

Two approaches could improve our understanding regarding the role of airway epithelium in allergic inflammation. *In vitro* co-culture of epithelial cells grown in ALI with one or more of the other immune cells that may play a role in allergic inflammation, such as DCs and T cells, in the presence of particular allergens may clarify the sequence of events leading to the development of allergic airway inflammation. These studies should be coupled with *in vivo* models utilizing airway epithelium-specific strains of knockout mice. These studies should start with tissue-specific knockouts of epithelial receptors interacting with allergens and continue with similar knockout strains of signaling molecules. These studies may reveal common links between major allergens for their interactions with the airway epithelium and improve our understanding of the basic mechanisms leading to allergen-specific sensitization and inflammation.

## Conflict of Interest Statement

The authors declare that the research was conducted in the absence of any commercial or financial relationships that could be construed as a potential conflict of interest.
